# Life course rural/urban place of residence, depressive symptoms and cognitive impairment among older adults: findings from the Longitudinal Aging Study in India

**DOI:** 10.1186/s12888-023-04911-9

**Published:** 2023-06-02

**Authors:** T. Muhammad

**Affiliations:** Department of Family & Generations, International Institute for Population Sciences, Mumbai, 400088 India

**Keywords:** Urban/rural residence, Depressive symptoms, Cognitive impairment, Older adults

## Abstract

**Background:**

Given the unique socioeconomic structures, and the rural/urban differentials in the prevalence of mental illnesses in the country, this study aimed to explore the associations of childhood, adulthood and late-life rural/urban place of residence with mental health outcomes, namely depressive symptoms and cognitive impairment, among older adults in India. The study also examined the relationship between older individuals’ life-course rural/urban place of residence and late-life mental and cognitive health.

**Methods:**

Utilizing data from the Longitudinal Aging Study in India (*n* = 28,027 older adults age 60 years and above), the study employed multivariable logistic and linear regression models to examine the association between urban/rural residential status, life-course residence, depressive symptoms and cognitive impairment among older adults.

**Results:**

Childhood and adulthood place of residence was not associated with depressive symptoms in older men and women. Current rural place of residence was positively associated with depressive symptoms in older women [adjusted odds ratio (aOR): 1.37, confidence interval (CI): 1.05–1.80] but not men. Childhood [aOR: 1.88, CI: 1.16–3.04], adulthood [aOR: 2.00, CI: 1.26–3.16] and current rural residence [aOR: 1.93, CI: 1.27–2.91] was positively associated with cognitive impairment in men. Only current rural residence [aOR: 1.71, CI: 1.29–2.27] was associated with cognitive impairment in women. There was no significant association between life-course place of residence and depressive symptoms except in case of lifetime rural residence Respondents with urban-urban-urban (childhood-adulthood-current) place of residence were less likely to have depressive symptoms [adjusted coefficient (aCoef.): -0.14, CI: -0.21- -0.07] compared to those with rural-rural-rural place of residence. There were significant associations between life-course residence and cognitive impairment except among rural-urban-rural and urban-rural-rural migrants, showing an urban advantage in cognitive function among older adults.

**Conclusions:**

This study showed significant associations between life-course residence and depressive symptoms among permanent rural/urban residents. The study also showed significant associations between life-course residence and cognitive impairment except among rural-urban-rural and urban-rural-rural migrants. Considering the rural disadvantage in mental and cognitive health among older adults, the government should continue to support policies that can improve access to education and healthcare among people residing in rural areas and women, in particular. The findings also urge social scientists and gerontologists in particular, to consider the importance of lifetime historical context while evaluating mental and cognitive health of older persons.

**Supplementary Information:**

The online version contains supplementary material available at 10.1186/s12888-023-04911-9.

## Background

The proportion of older population in India is expected to increase from 9.4% in 2017 (126 million), to 19.1% in 2050 (317 million) [1]. With the increase in the proportion of the ageing population, the health risks accumulated over the life course are also increasing. Studies among older adults in the Indian context have shown that sedentary lifestyles were associated with an increase in the risk of obesity and resultant cardiovascular diseases and disability, especially in urban areas [2, 3]. The intensive care needs and long- term support for older adults with poor mental health and reduced cognition can burden the healthcare system in India significantly as this population continues to grow.

With growing urbanization, people are exposed to large number of social, physical and environmental risk factors in urban areas that contribute to their increased stress, which in turn, deteriorates their mental health [4]. By contrast, urban centers provide better access to education, highly paid jobs and health care facilities that result in increased treatment seeking [5, 6]. Further,  a longitudinal study suggested that availability of physical and health resources and facilities associated with lifetime residential status in urban areas may lead to mental health improvement in later life among urban residents than rural counterparts [7]. The balance between the risk factors and protective factors of mental health associated with rural/urban residence calls for a better understanding of the interactions between life-course residential status and mental health. Similarly, multiple studies have shown that migration from one residential area (rural or urban) to another area is an important factor that could impact later-life cognitive function [8–10]. Most of the migration from one place to another mostly happens during adulthood; males tend to migrate for employment-related reasons, whereas females migrate predominantly due to marriage and family-related reasons [11, 12].

After moving from a rural area to an urban area at any stage of life, multiple factors at individual, family and community levels might contribute to improving or worsening mental and cognitive health of people in their later life. For example, rural to urban migration after entering to adulthood may reduce the quality relationships which can limit social interactions and cognitive stimulation among adults, and the resultant cumulative stress can undermine mental health and cognitive function in the long term [7, 13]. On the other hand, according to the healthy migrant phenomenon, those who migrate tend to be healthier and potentially less vulnerable to adverse health effects than those who do not migrate [14]. Moreover, those who migrate to urban areas may achieve better socioeconomic position, work and living conditions and access to healthcare [15–17], and therefore lower levels of mental disorders compared to people who reside in rural areas. Besides, urban non-migrants may have higher chances of improved mental health as they experience the beneficial aspects of the urban environment such as higher levels of education and income that allow them to maintain good health and wellbeing and avoid potential stressor of migration [18].

Recent studies have explored the socioeconomic factors and wealth-based inequalities associated with depression and cognitive impairments of older people in developing countries [19–23], and found that the socioeconomic structures, the pattern of rural/urban inequality and associated mental health outcomes in India are not similar to what is previously tested in Western societies. Therefore, there is a substantial knowledge gap regarding depressive symptoms and cognitive impairment among the rapidly growing older people with different status of rural/urban place of residence in India. This study aimed to explore the associations of childhood, adulthood and late-life place of residence with mental health outcomes (depressive symptoms and cognitive impairment) among older Indians. Guided by a life course perspective, the study also examined the relationship between older individuals’ life-course place of residence and late-life mental and cognitive health in India.

## Methods

### Data

This study used data from the first wave of the Longitudinal Aging Study in India (LASI, 2017-19). The Harvard T.H. Chan School of Public Health, the International Institute for Population Sciences (IIPS), and the University of Southern California (USC) collaborated on data collection procedures of the LASI survey. The nationally representative longitudinal survey is proposed to collect crucial information on the physical, social, and cognitive well-being of India’s older citizens over a 25-year period. The baseline survey of LASI collected data of over 72,000 people age 45 and over, as well as their spouses (of any age), across India’s states and union territories. The sample is based on a multistage stratified cluster sample design that includes three and four separate phases of rural and urban region selection, respectively. The LASI report contains information on sample design, survey instruments, fieldwork, data collecting and processing, and response rates [24]. The details of the survey strategies are also described elsewhere [25]. The final sample for the current study included 28,027 (14,286 males and 13,741 females) older Indian adults age 60 years or older with information on formal residence during childhood and adulthood and late life residence (at present).

### Measures

#### Outcome variables

Major probable depression as an outcome variable of this study was coded as 0 for not having depressive symptoms and 1 for having depressive symptoms. Probable depression among older adults with symptoms of dysphoria, was calculated using the Short Form Composite International Diagnostic Interview (CIDI-SF) [26, 27]. This scale estimates a probable psychiatric diagnosis of major depression and has been validated in field settings [28, 29]. It has 3 screening and 7 symptom-based questions and a score of three or more on a scale of 0–10 leads to a 0.55 probability of CIDI caseness of major depression [30], which in this study is labelled as having depressive symptoms. The scale was validated with well-established cross-cultural applicability especially by non-clinicians in general population surveys and widely used in population-based health surveys [29, 31, 32]. Cronbach’s alpha indicated that CIDI-SF has acceptable reliability (α = 0.7). The scale was used in continuous form as the outcome, modelled as the number of depressive symptoms, during the analysis of life-course residential status and depressive symptoms.

Cognitive impairment was another outcome variable of this study and was measured through five broad domains (memory, orientation, arithmetic function, executive function, and object naming). Memory was measured using immediate word recall, delayed word recall; orientation was measured using time and place measure; arithmetic function was measured through backward counting, serial seven, and computation method; executive function was measured through paper folding and pentagon drawing method, and object naming was also done to measure the cognitive impairment among older adults. The overall score ranged between 0 and 43, and a higher score indicated better cognitive functioning. The lowest 10th percentile was used as a proxy measure of poor cognitive functioning [28]. Further, during the analysis of life-course residential status and cognitive impairment, the score was reversed to assess the cognitive impairment among older adults and thus after reversing, the higher score indicated higher levels of cognitive impairment.

#### Exposure variables

Current place of residence was coded as urban (those residing in statutory/Census towns) and rural (those residing in villages). Further, considering information on their formal residence, respondents were classified as urban or rural residents according to their residence during childhood and adulthood if they reported living in either town or village locations in response to survey questions on where they spent most of their childhood (up to age 14) and most of their adult life. Additionally, those who responded to the question “How many years have you been living (continuously) in this area?” as “since birth”, were accorded a childhood/ adulthood place of residence respective to their current place of residence. Further, categories of lifetime residence were created using a sequence of childhood, adulthood, and current time periods with responses classified as urban or rural. This was based on the survey questions on where individuals spent most of their childhood and most of their adult life, along with the location of their current household residence, and resulted in 8 possible life-course patterns: 1) rural-rural-rural; 2) rural-rural-urban; 3) rural-urban-urban; 4) rural-urban-rural; 5) urban-rural-rural; 6) urban-urban-rural; 7) urban-rural-urban and 8) urban-urban-urban. The same approach was followed in previous studies [33, 34].

### Other covariates

Independent variables considered for adjustment in the analysis included sex (male/female); age (60–69, 70–79, and 80 + years); education (no formal education, primary, secondary and higher); work status (never worked, worked but currently not, currently working and retired) marital status (currently married, widowed and others which included separated, divorced and never married); and living arrangements (living alone, with spouse, with spouse and children and with others). Further, to control for the possible confounding of physical health in the association of residential status with mental health outcomes, self-rated health and functional difficulty were included in the analysis and self-rated health was coded as good which includes very good, good and fair whereas, poor includes poor and very poor. Difficulty in activities of daily living and instrumental activities of daily living (ADL and IADL) were categorised into yes if the respondent reported at least one difficulty in the daily living basic and instrumental activities, respectively [35].

Besides, the following socio-demographic variables were selected and included in the analysis as per literature. Monthly per capita consumption expenditure (MPCE) quintile was assessed using household consumption data and was divided into five quintiles of poorest, poor, middle, rich and richest [24]. Religious group was coded as Hindu, Muslim, Christian, and Others. Caste group was recoded as Scheduled Caste/Scheduled Tribe (SC/ST), Other Backward Classes (OBC), and others [24]. Others refer to those with higher social status, mostly belonging to the higher socioeconomic groups and upper castes [36]. Finally, the regions of the country were categorised into North, Central, East, Northeast, West, and South.

### Statistical analysis

Descriptive statistics were reported at the initial stage. Bivariate analysis (cross-tabulations) was conducted to report the prevalence of depressive symptoms and cognitive impairment among the study participants. Mean scores of depressive symptoms and cognitive impairment along with their confidence intervals (CI) were reported across the eight categories of life-course residence. For analyzing the statistical significance of the associations for each category, t-test was conducted and p-values are reported. Multivariable logistic regression models were employed to examine the unadjusted and adjusted (model 1 adjusted for age, education and work status model 2 adjusted for all the covariates included in this study) associations of urbanicity of residence in childhood and adulthood as well as late life with depressive symptoms and cognitive impairment.

Further, linear regression models were employed to examine the association of life-course residential status (with eight possible categories) with depressive symptoms and cognitive impairment (continuous outcomes) adjusting for all the covariates included in this study. Unadjusted and adjusted odds ratios (uOR and aOR) and coefficients (uCoef and aCoef) along with beta coefficients are presented along with 95% CI. Individual weights were applied during the analysis to account for the clustered and stratified survey design. The values of area under the receiver operating characteristic curve (AUROC) of 0.7 and 0.8 for the outcomes of depressive symptoms and cognitive impairment in the logistic regressions (supplementary material Figure S1a and b) suggested good fit models. Also, linear regressions met all assumptions and included tests for linearity and normality of residuals and multi-collinearity of predictor variables and are presented through visual inspections of scatterplots, quantile-quantile plots and variance inflation factors (VIFs) (supplementary material Figures S2a- S3b and Table S1, respectively). All the analysis was conducted in Stata version 15.1.

## Results

Descriptive statistics of older men and women in this study are displayed in Table 1. A little more than half of the sample population was males (51.37%) and a total of 16.6% of older men and 55.02% of older women were widowed. A total of 2.54% older men and 9.01% older women lived alone. More than 61% and 85% of older women had no or primary level of formal education. A proportion of 22.16% and 26.13% of older men and women had poor self-rated health, respectively. Notably, 26.68% of older men and 32.2% of older women currently resided in urban areas. On the other hand, 4.3% of rural-dwelling older adults and 6.26% urban-dwelling older adults were living alone. A total of 22.16% of older men and 26.13% of older women as well as 21.48% of rural-dwellers and 25.17% of urban-dwellers reported a poor self-rated health in this study.

**Table 1:** Socio-economic and health profile of older adults, Longitudinal Study of Aging in India, 2017-19 (*n* = 28,027)

Background Factors	Men (%)	Women (%)	Rural (%)	Urban (%)	Total (%)
**Age (in years)**					
60–69	8552 (58.14)	8439 (58.65)	5817 (58.08)	11,174 (58.52)	16,991 (58.39)
70–79	4260 (30.84)	3813 (29.68)	2782 (31.45)	5291 (29.79)	8073 (30.28)
80+	1474 (11.02)	1489 (11.66)	903 (10.47)	2060 (11.69)	2963 (11.33)
**Sex**					
Men			4567 (46.68)	9719 (53.32)	14,286 (51.37)
Women			4935 (53.32)	8806 (46.68)	13,741 (48.63)
**Marital status**					
Currently in union	11,730 (80.98)	6217 (42.9)	5911 (59.01)	12,036 (63.9)	17,947 (62.46)
Widowed	2170 (16.6)	7143 (55.02)	3240 (38.48)	6073 (33.95)	9313 (35.28)
Others	386 (2.42)	381 (2.09)	351 (2.51)	416 (2.15)	767 (2.26)
**Living arrangement**					
Alone	345 (2.54)	1091 (9.01)	424 (4.3)	1012 (6.26)	1436 (5.69)
With spouse	3550 (26.11)	2036 (14.81)	1701 (18.03)	3885 (21.69)	5586 (20.61)
Others	10,391 (71.35)	10,614 (76.19)	7377 (77.67)	13,628 (72.05)	21,005 (73.7)
**Educational status**					
No education	8430 (61.44)	11,583 (85.09)	5129 (51.87)	14,884 (81.69)	20,013 (72.94)
Primary	4007 (26.15)	1611 (11.79)	2714 (30.91)	2904 (14.29)	5618 (19.17)
Secondary/higher	1849 (12.42)	547 (3.12)	1659 (17.21)	737 (4.02)	2396 (7.9)
**Self-rated health**					
Good	11,059 (77.84)	10,087 (73.87)	7182 (78.52)	13,964 (74.83)	21,146 (75.9)
Poor	2924 (22.16)	3359 (26.13)	2111 (21.48)	4172 (25.17)	6283 (24.1)
**Wealth quintile**					
Poorest	2893 (21.02)	2836 (22.29)	1996 (22.33)	3733 (21.35)	5729 (21.64)
Poorer	2905 (21.31)	2808 (21.43)	1940 (19.78)	3773 (22.03)	5713 (21.37)
Middle	2865 (21.26)	2802 (20.1)	1895 (20.66)	3772 (20.71)	5667 (20.7)
Richer	2832 (19.22)	2704 (19.12)	1846 (19.59)	3690 (19)	5536 (19.17)
Richest	2791 (17.19)	2591 (17.06)	1825 (17.65)	3557 (16.91)	5382 (17.13)
**Religion**					
Hindu	10,482 (81.95)	9836 (82.03)	6671 (78.64)	13,647 (83.38)	20,318 (81.99)
Muslim	1717 (11.76)	1661 (10.79)	1585 (15.29)	1793 (9.63)	3378 (11.29)
Others	2087 (6.29)	2244 (7.18)	1246 (6.07)	3085 (6.99)	4331 (6.72)
**Caste**					
SC/ST	4638 (26.53)	4598 (26.47)	2123 (13.79)	7113 (31.78)	9236 (26.5)
OBC	5545 (46.63)	5191 (46.9)	3678 (49.16)	7058 (45.77)	10,736 (46.76)
Others	4103 (26.84)	3952 (26.63)	3701 (37.05)	4354 (22.45)	8055 (26.74)
**Place of residence**					
Urban	4567 (26.68)	4935 (32.2)			9502 (29.36)
Rural	9719 (73.32)	8806 (67.8)			18,525 (70.64)
**Region**					
North	2675 (12.45)	2623 (13.51)	1823 (12.31)	3475 (13.24)	5298 (12.97)
Central	2075 (22.88)	1644 (18.64)	749 (13.86)	2970 (23.72)	3719 (20.82)
East	2674 (23.95)	2105 (20.34)	1074 (13.02)	3705 (26.01)	4779 (22.19)
Northeast	1611 (2.84)	1739 (3.36)	803 (2.08)	2547 (3.51)	3350 (3.09)
South	3403 (21.85)	3692 (27.55)	3209 (35.13)	3886 (20.26)	7095 (24.62)
West	1848 (16.02)	1938 (16.6)	1844 (23.61)	1942 (13.27)	3786 (16.3)

Counts are unweighted; w%: percentages weighted to account for the complex survey design and to provide the national population estimates

*SC/ST* Scheduled caste/scheduled tribe, *OBC* Other backward classes

**Fig. 1:**
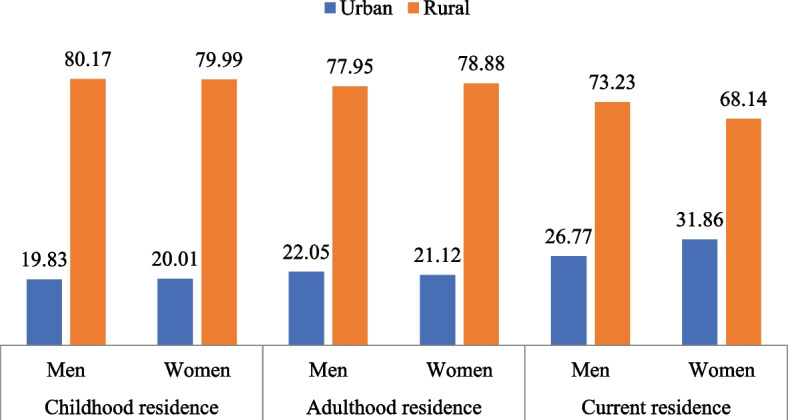
Percentage distribution of older adults by childhood, adulthood and current residence

**Fig. 2:**
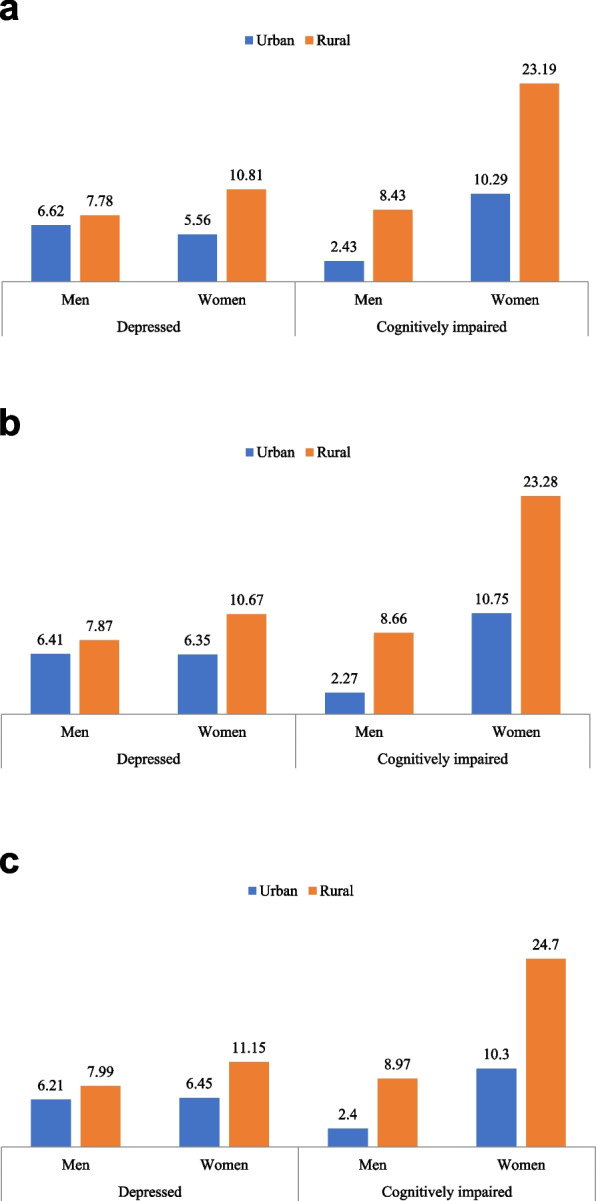
**a** Prevalence of depressive symptoms and cognitive impairment by childhood residence. **b** Prevalence of depressive symptoms and cognitive impairment by adulthood residence. **c** Prevalence of depressive symptoms and cognitive impairment by current residence

Figure 1 presents the percentage distribution of older adults by childhood, adulthood and current residence. Further, among the male and female sample with urban childhood residence, the rate of depressive symptoms was 6.62% and 5.56%, while the rate among older adults with rural childhood residence was 7.78% and 10.81% in men and women respectively. Similarly, the rate of cognitive impairment was substantially higher among women than men with only 2.43% and 10.29% of older men with rural and urban childhood residence having cognitive impairment against 8.43% and 23.19% of older women with respective childhood residence having cognitive impairment (Fig. 2a). The rates of depressive symptoms in the samples based on type of adulthood residence were 6.41% and 6.35% for urban and 7.87% and 10.67% for rural residence. Also, the rate of cognitive impairment among men and women with rural residence in adulthood was 8.66% and 23.28% and with urban residence only 2.27% and 10.75%, respectively (Fig. 2b). By current place of residence, higher percent of men (7.99%) and women (11.15%) residing in rural areas had depressive symptoms compared to those in urban areas (6.21% and 6.45%), and higher percent of rural residing men (8.97%) and women (24.7%) had cognitive impairment than their counterparts in urban areas (2.4% and 10.3%) (Fig. 2c).

There were no significant associations between childhood and adulthood residence with depressive symptoms among older men in this study (Table 2). However, in the unadjusted logistic regression model, the odds for men who reside in rural areas in their late life were 1.28 (95% CI: 1.02–1.61), which was higher in comparison to men who reside in urban areas. The association was insignificant after adjusting for the selected covariates in the study. For female counterparts, there was significant positive association of childhood rural residence (aOR: 1.47, 95% CI: 1.04–2.07) and current rural residence (aOR: 1.37, 95% CI: 1.05–1.80) with depressive symptoms after adjusting for all the covariates. In case of adulthood residence, the odds of depressive symptoms after adjusting for age, education and work status were 1.44 times (95% CI: 1.05–1.96) higher among women who spent most of their childhood in rural areas in comparison to women who spent most of their childhood in urban areas.

**Table 2:** Logistic regression estimates of depressive symptoms and cognitive impairment by childhood, adulthood and current residence

Variables	Depressive symptoms			Cognitive impairment		
	Unadjusted model uOR (95% CI)	Model 1 aOR (95% CI)	Model 2 aOR (95% CI)	Unadjusted model uOR (95% CI)	Model 1 aOR (95% CI)	Model 2 aOR (95% CI)
**Men**						
** Childhood residence**						
Urban	Ref.	Ref.	Ref.	Ref.	Ref.	Ref.
Rural	1.19 (0.92–1.54)	1.14 (0.88–1.48)	0.94 (0.72–1.22)	3.69*** (2.37–5.75)	2.02** (1.26–3.24)	1.88* (1.16–3.04)
** Adulthood residence**						
Urban	Ref.	Ref.	Ref.	Ref.	Ref.	Ref.
Rural	1.25 (0.97–1.60)	1.20 (0.93–1.55)	0.98 (0.76–1.26)	4.08*** (2.66–6.27)	2.13** (1.35–3.36)	2.00** (1.26–3.16)
** Current residence**						
Urban	Ref.	Ref.	Ref.	Ref.	Ref.	Ref.
Rural	1.28* (1.02–1.61)	1.24 (0.97–1.57)	0.95 (0.74–1.22)	4.22*** (2.90–6.16)	2.09*** (1.40–3.12)	1.93** (1.27–2.91)
**Women**						
** Childhood residence**						
Urban	Ref.	Ref.	Ref.	Ref.	Ref.	Ref.
Rural	2.06*** (1.49–2.85)	1.68** (1.20–2.35)	1.47* (1.04–2.07)	2.63*** (1.80–3.85)	1.23 (0.84–1.80)	1.25 (0.85–1.85)
** Adulthood residence**						
Urban	Ref.	Ref.	Ref.	Ref.	Ref.	Ref.
Rural	1.76*** (1.31–2.36)	1.44* (1.05–1.96)	1.33 (0.97–1.81)	2.52*** (1.81–3.50)	1.29 (0.91–1.82)	1.35 (0.95–1.92)
** Current residence**						
Urban	Ref.	Ref.	Ref.	Ref.	Ref.	Ref.
Rural	1.93*** (1.52–2.44)	1.61*** (1.25–2.09)	1.37* (1.05–1.80)	2.95*** (2.28–3.81)	1.58** (1.19–2.09)	1.71*** (1.29–2.27)

*uOR* unadjusted Odds ratio, *aOR* adjusted Odds ratio, *CI* Confidence interval; Model 1 is adjusted for age, education and work status; Model 2 is additionally adjusted for marital status, living arrangement, self-rated health, difficulty in activities of daily living, difficulty in instrumental activities of daily living, monthly per capita consumption expenditure, religion, caste and country regions; *Ref.* Reference category

*p < 0.05, **p < 0.01, ***p < 0.001

In case of cognitive impairment, childhood (aOR: 1.88, 95% CI: 1.16–3.04), adulthood (aOR: 2.00, 95% CI: 1.26–3.16) and current (aOR: 1.93, 95% CI: 1.27–2.91) rural residences were independently associated with cognitive impairment among older men. Similarly, current rural residence was positively associated with cognitive impairment among older women (aOR: 1.71, 95% CI: 1.29–2.27). However, in case of older women, after controlling for all the covariates, there was no significant but positive associations of childhood (aOR: 1.25, 95% CI: 0.85–1.85) and adulthood (aOR: 1.35, 95% CI: 0.95–1.92) rural residence with cognitive impairment.

Table 3 presents the depressive symptoms and cognitive impairment among older adults in the study by their life-course place of residence. A higher percentage of the sample (69.26%) had a lifetime rural place of residence (rural childhood, rural adulthood and rural current place of residence). On the other hand, 18.19% of the sample population had a lifetime urban place of residence (urban childhood, urban adulthood and urban current place of residence). Further, older adults who had a rural childhood, urban adulthood and rural late-life place of residence had higher mean score of depressive symptoms compared to all other categories. However, older adults who had an urban childhood, rural adulthood and rural late-life place of residence had higher mean score of cognitive impairment compared to all other categories.

**Table 3:** Distribution of older adults by life course residence and rates of depressive symptoms and cognitive impairment

Variables	Sample (w%)	Mean depressive symptoms (CI)	t-test *p*-value	Mean cognitive impairment (CI)	t-test *p*-value
**Life course residence**					
Rural-rural-rural	18,126 (69.26)	0.96 (0.92-10)	< 0.001	22.95 (22.85–23.06)	< 0.001
Rural-rural-urban	2,980 (8.14)	0.75 (0.67–0.83)	0.002	20.78 (20.54–21.03)	< 0.001
Rural-urban-urban	831 (2.45)	0.69 (0.54–0.84)	0.316	19.54 (19.11–19.98)	< 0.001
Rural-urban-rural	76 (0.25)	1.79 (0.97–2.60)	0.199	20.46 (18.95–21.98)	0.127
Urban-rural-rural	102 (0.44)	0.95 (0.50–1.41)	0.218	23.91 (22.54–25.27)	0.114
Urban-urban-rural	221 (0.71)	1.21 (0.82–1.60)	0.970	20.76 (19.81–21.71)	0.070
Urban-rural-urban	40 (0.56)	0.03 (-0.10-0.17)	0.253	18.04 (17.48–18.61)	0.027
Urban-urban-urban	5,651 (18.19)	0.62 (0.57–0.67)	< 0.001	18.67 (18.50-18.84)	< 0.001
**Total**	28,027 (100)	0.86 (0.83–0.89)		21.97 (21.86–22.02)	

Counts are unweighted; w%, weighted percentages to account for the complex survey design and to provide the national population estimates; Depressive symptoms on a scale of 0-10 and cognitive impairment on a scale of 0-43

*CI* Confidence interval

Table 4 presents the adjusted linear regression estimates of depressive symptoms by life-course place of residence among older adults. Older adults who had a lifetime urban place of residence (urban childhood, urban adulthood and urban current place of residence) had significantly lower likelihood of depressive symptoms (aCoef: 0.14, *p* < 0.001) than those who had lifetime rural place of residence.

**Table 4:** Multivariate linear regression estimates of depressive symptoms among older adults by their life course residence

Variables	uCoef (95% CI)	Beta	aCoef (95% CI)	Beta
**Life course residence**				
Rural-rural-rural	Ref.	Ref.	Ref.	Ref.
Rural-rural-urban	-0.18*** (-0.27 - -0.10)	-0.03	-0.12** (-0.21 - -0.03)	-0.02
Rural-urban-urban	-0.14 (-0.30–0.01)	-0.01	-0.08 (-0.24–0.07)	-0.01
Rural-urban-rural	0.26 (-0.24–0.75)	0.01	0.24 (-0.25–0.73)	0.01
Urban-rural-rural	0.20 (-0.23–0.63)	0.01	0.12 (-0.30–0.55)	0.00
Urban-urban-rural	-0.07 (-0.36–0.22)	-0.00	-0.04 (-0.33–0.25)	-0.00
Urban-rural-urban	0.33 (-0.35–1.01)	0.01	0.37 (-0.32–1.05)	0.01
Urban-urban-urban	-0.23*** (-0.30 - -0.16)	-0.04	-0.14*** (-0.21 - -0.07)	-0.02

*uCoef* unadjusted Coefficients, *CI* Confidence interval, *aCoef* Coefficients adjusted for age, education and work status, marital status, living arrangement, self-rated health, difficulty in activities of daily living, difficulty in instrumental activities of daily living, monthly per capita consumption expenditure, religion, caste and country regions, *Beta* Standardized beta coefficients; Life-course categories represent childhood-adulthood-current residence, *Ref.* Reference category

*p < 0.05, **p < 0.01, ***p < 0.001

Table 5 presents the adjusted linear regression estimates of cognitive impairment by life-course place of residence among older adults. Older adults who had a lifetime urban place of residence (urban childhood, urban adulthood and urban current place of residence) had significantly lower likelihood of cognitive impairment (aCoef: 2.34, *p* < 0.001) than those who had lifetime rural place of residence.

**Table 5:** Multivariate linear regression estimates of cognitive impairment among older adults by their life course residence

Variables	uCoef (95% CI)	Beta	aCoef (95% CI)	Beta
**Life course residence**				
Rural-rural-rural	Ref.	Ref.	Ref.	Ref.
Rural-rural-urban	-2.54*** (-2.81 - -2.26)	-0.12	-1.77*** (-2.00 - -1.53)	-0.08
Rural-urban-urban	-3.25*** (-3.72 - -2.78)	-0.09	-1.65*** (-2.04 - -1.27)	-0.04
Rural-urban-rural	-2.57** (-4.12 - -1.03)	-0.02	-0.47 (-1.72–0.79)	-0.00
Urban-rural-rural	-0.17 (-1.55–1.20)	-0.00	-1.03 (-2.14–0.08)	-0.01
Urban-urban-rural	-2.20*** (-3.12 - -1.28)	-0.03	-1.09** (-1.84 - -0.35)	-0.01
Urban-rural-urban	-3.90*** (-6.10 - -1.70)	-0.02	-2.38** (-4.16 - -0.59)	-0.01
Urban-urban-urban	-4.49*** (-4.70 - -4.28)	-0.27	-2.34*** (-2.52 - -2.15)	-0.14

*uCoef* unadjusted Coefficients, *CI* Confidence interval, *aCoef* Coefficients adjusted for age, education and work status, marital status, living arrangement, self-rated health, difficulty in activities of daily living, difficulty in instrumental activities of daily living, monthly per capita consumption expenditure, religion, caste and country regions, *Beta* Standardized beta coefficients; Life-course categories represent childhood-adulthood-current residence *Ref.* Reference category

*p < 0.05, **p < 0.01, ***p < 0.001

## Discussion

The study observed significant differences in depressive symptoms and cognitive impairment among older Indians by their urban/rural residential status. Rural place of residence was a significant risk factor for depressive symptoms among older women and cognitive impairment among older men and women in this study. Again, older adults who resided in urban areas during childhood, adulthood and later life had the lowest level of depressive symptoms and cognitive impairment; whereas those who resided in rural areas during their lifetime had the highest level of depressive symptoms and cognitive impairment among the eight groups. These findings remained after adjusting for the socio-demographic and economic factors such as age, education, work status, household consumption quintiles, religion, caste and regions as well as psychosocial resources such as marital status and living arrangements and self-rated health and functional difficulties, although they accounted for much of the associations.

Previous studies among different populations in different age groups have shown that urban/rural residential status of individuals has significant association with their health and wellbeing [37, 38]. A study among adolescents in West Africa suggests that schooling and urban residence may have positive effects on their behaviors and skills which in turn result in the improvement of cognitive abilities [39]. Another study among the general population in Indonesia found that social capital increases life satisfaction among urban residents but not rural residents [40]. Also, older adult social participation was shown to have differential effects on health in rural and urban areas [41]. The higher rates of depression and cognitive impairment in rural areas in this study may be explained by the fact that the rural population is engaged in highly stressful jobs, have lack of formal education, inadequate healthcare and housing facilities, and perform worse in other social determinants of health. Previous studies have also reported a similar urban-rural gradient in depression and cognitive function among older adults in India, with a rural disadvantage [42–45]. Multiple studies in other countries have however, reported positive, negative and no effect of rural residence on depression in later life [46, 47]. The current analysis showed no significant association of place of residence at any stage of life with depressive symptoms among older men. The current finding is consistent with a study in Ghana that found no significant association between childhood, adulthood and late life urbanicity with depression [48]. This is also in line with an earlier study suggesting that current rural residence is more important in predicting depression than a prior history of a rural residence [33].

However, the current analysis showed that older women who resided in rural areas during their childhood are more likely to develop depressive symptoms than older women who lived their childhood in urban communities. The association remained significant even after adjusting for all the covariates in the study. Although the association is of borderline statistical significance, the finding may be explained by increased stress among people with limited resource in village areas. Separating from one’s biological family may in turn cause greater levels of stress, lower levels of social cohesion, and the loss of social support that could adversely affect cognitive function [49]. The different levels of depressive symptoms and cognitive impairment among rural resident older men and women may be explained by differential impact of lack of social networks, food insecurity and unequal gender norms on mental health of men and women in rural areas [50]. This may also be explained by the limited access to education and healthcare as well as lower rate of labor force participation in rural settings particularly among women [43, 51].

On the other hand, the positive association of rural residence during adulthood with depressive symptoms disappeared after adjusting for socioeconomic characteristics. Notably, the effect that is not seen in models adjusting for potential confounding factors may suggest that other factors directly related to mental health might have also contributed to influence the finding. For instance, lack of income, long distance to health facilities, and limited services and amenities may lead to increased risk of depressive symptoms among older people who reside in rural areas. However, as documented in previous studies, the factors that potentially reduce the risk of mental illnesses in rural areas may include residential stability, quality relationships and stable social networks. Again, individual factors such as resilience, independence and self-sufficiency may reduce the risk of depressive symptoms and cognitive impairment among rural residents. These could be considered while developing future policies and programs for older Indian adults.

Furthermore, considering the life-course residential patterns, the association between place of residence and mental health outcomes in this study is only significant in case of older people who stayed in the same residential area and those who resided in urban areas throughout their life had lower chances of depressive symptoms or cognitive impairment. On the other hand, people who resided in rural areas during childhood and adulthood and migrated to urban areas had less likelihood of depressive symptoms and cognitive impairment in this study compared to rural non-migrants. This is in line with multiple cross-sectional as well as longitudinal studies on the positive effects of rural-to-urban migration on mental health of older adults [4, 47]. The finding also supports the “healthy migrant” hypothesis, which states that migrants represent a positively selected group of individuals with respect to health, relative to the general population in origin societies [52, 53]. However, the current finding is in contrary to a previous study in China that suggested that migration from rural to urban areas may lead to experience of social stigma, discrimination and inequity, which ultimately result in social exclusion and negatively affect the mental health of migrant people compared to non-migrant people [54].

Interestingly, unlike depressive symptoms, the associations of life-course residential status with cognitive impairment were highly significant in this study. Compared to non-migrant rural residents, those who moved to urban areas during adulthood or late life were less likely to have cognitive impairment. The underlying mechanism by which residential changes may influence cognitive function is not well established yet. Health and wellbeing of a person is influenced by the complex interactions between environmental factors and body functions and structures as well as activities and participation [55, 56]. As people age, functional or intrinsic capacities, like walking, hearing, seeing, and cognitive ability will reduce and this is escalated in poor socioeconomic setting [57, 58]. Besides, people’s health conditions and adaptability change as they move in and out of different neighborhood over the life course [59, 60]. Studies have reported that residential mobility and migration are associated with an increased economic instability and changes in marital status, family composition and employment [61], which may gradually contribute to cognitive reserve and improved cognitive abilities among older people. In addition, the psychological and cognitive health is improved by the social participation among older people facilitated by the urban community environment [62, 63].

On the other hand, people from rural areas who resided in urban areas during adulthood had lower chances of cognitive impairment than rural non-migrants. Similarly, non-migrant urban residents were less likely to have cognitive impairment in this study. This is supportive of earlier findings that urban residence is positively associated with cognitive function among older adults, attributed to several reasons such as higher literacy, educational and occupational opportunities and health resources [64]. Similarly, urban environment is considered as psychologically and socially demanding [65], which may positively affect the cognitive reserve among older urban residents. Further, the lifetime urban residence might also include those who migrate from one urban area to another during their life time. Consistently, moving from one urban area to another urban area may reflect an increase in socioeconomic standing, such as education and income [12]. The higher magnitude of the association of life-time urban residents with better cognition than those who moved to urban areas during adulthood or late life suggest that the rural-to-urban migrants may have lower access to utilization of health care and limited social support than urban residents [66–68]. This is again reflected in the reduced odds after accounting for socioeconomic characteristics in the fully adjusted analysis. The finding, thus, suggest that, the differences may be largely due to the socioeconomic disadvantages among rural-to-urban migrants compared with urban residents [69]. Nevertheless, specific environmental effects independent of socioeconomic conditions on mental and cognitive health need further exploration including the effect of population density, green areas, noise and diet.

The study covers a large sample of older adults from a geographically large and socioeconomically heterogeneous country. Moreover, the findings are robust as the analysis employed multiple linear regression models after adjusting for a large number of potential confounders. Furthermore, during the data collection, the LASI used reliable valid measures and trained interviewers gathered the data. Nevertheless, there are several limitations to be noted. Without knowing the reasons for and/or implications of relocating from urban-to-rural areas in India, it is unclear whether and how this form of migration is associated with cognitive function. Further, in the absence of indicators of reasons for migration, the migration variable used in this study may not have adequately captured all aspects of people’s migration history. Although the study included many of the possible confounding factors that may relate to cognition, additional unmeasured factors such as genetic factors, occupational status, environmental pollution, and healthcare utilization may have contributed to the associations [70]. Similarly, length of stay in rural or urban areas may have differential impact on cognitive function among older persons [71, 72], which is not accounted in this study. Further, the sample size of some groups of life-course residential categories was small (e.g., rural-urban-rural migrants) which might have influenced the study findings. Lastly, due to the cross-sectional nature of the analysis, interpretation of the results requires caution. Additional longitudinal studies are required to further examine these and other factors that may contribute to migration-related differences in mental health and cognitive function. Another limitation is the difficulty accounting for different rural factors (population density, distance to services, economic base, etc.), which may affect depressive symptoms and cognitive abilities.

The findings are crucial in terms of its policy implications. Taken together, effective interventions should be developed and implemented in rural areas to reduce the higher rate of depressive symptoms and prevent or prolong cognitive decline in older adults, especially among those who are life time rural-dwellers. To that end, health care providers should promote and employ early screening for probable depression and cognitive impairments among older adults living in rural areas and among rural resident women in particular and identify at-risk subpopulations. The current findings based on the life course residence indicating the differential impact of transitions in rural/urban residence across different stages of life on mental health and wellbeing add to the knowledge gap in environmental influence on older adults’ healthy and active aging, especially in low- and middle-income country settings. This study also calls for further attention to future research on the mind sponge mechanism suggesting that individuals’ wellbeing is negatively influenced by their exposure to multiple contextual risk factors such as lack of health awareness and limited healthcare services, particularly in rural areas [73, 74].

## Conclusions

This study showed significant associations between life-course residence and depressive symptoms among permanent rural/urban residents. The study also showed significant associations between life-course residence and cognitive impairment except among rural-urban-rural and urban-rural-rural migrants. Current findings are important for policy-makers and clinicians. Considering the rural disadvantage in mental and cognitive health among older adults, the government should continue to support policies that can improve access to education and healthcare among people residing in rural areas and women, in particular. Clinicians working in rural areas should be aware that their patients have an increased risk of developing mental illnesses such as depression and cognitive impairment than people in urban centres. Besides, there are considerable barriers in the delivery of care to older adults with depressive symptoms in rural areas. For individuals residing in rural areas, access to primary and secondary care, as well as access to social and psychological services may be limited by travel distance, lack of service providers, and cultural factors, which can increase their risk of mental illnesses and cognitive decline. Thus, models of care for older adults with depressive symptoms and cognitive impairment require further research with special attention to rural areas. The findings also urge social scientists and gerontologists, in particular, to consider the importance of lifetime historical context while evaluating mental and cognitive health of older persons.

## Supplementary Information

**Additional file 1: Figure S1a and b.** The receiver operating characteristic (ROC) curves with respective area under the curve (AUC). **Figure S2a and b.** Plots of residuals against fitted values (close to zero variation of mean of the residuals (y-axis) against fitted values (x-axis) of predictors suggests no violation of linearity). **Figure S3a and b.** Quantile-quantile plots showing the normality of the residuals. **Table S1.** VIF estimates for the selected explanatory variables.

## Data Availability

The data are available at The Gateway to Global Aging Data (https://g2aging.org/?section=overviews&study=lasi).
