# Supplementary material for: Life course rural/urban place of residence, depressive symptoms and cognitive impairment among older adults: findings from the Longitudinal Aging Study in India
Source: BMC Psychiatry. 2023 Jun 2;23:391. doi: 10.1186/s12888-023-04911-9 (PMC10239177; doi:10.1186/s12888-023-04911-9)
Supplement: Supplementary file 1 — Additional file 1: Figure S1a and b. The receiver operating characteristic (ROC) curves with respective area under the curve (AUC). Figure S2a and b. Plots of residuals against fitted values (close to zero variation of mean of the residuals (y-axis) against fitted values (x-axis) of predictors suggests no violation of linearity). Figure S3a and b. Quantile-quantile plots showing the normality of the residuals. Table S1. VIF estimates for the selected explanatory variables. [file 12888_2023_4911_MOESM1_ESM.pdf]

Figures S1a and S1b: The receiver operating characteristic (ROC) curves with respective area under the curve (AUC)

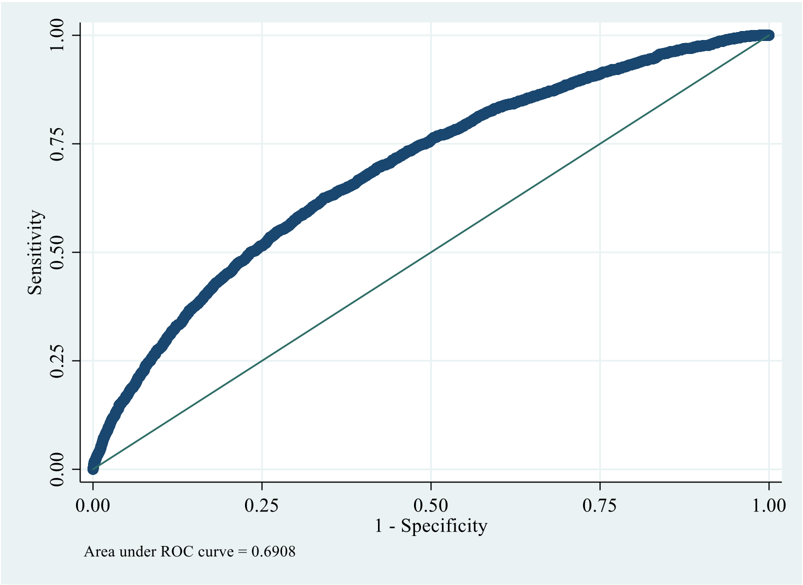

a; Outcome: Depressive symptoms

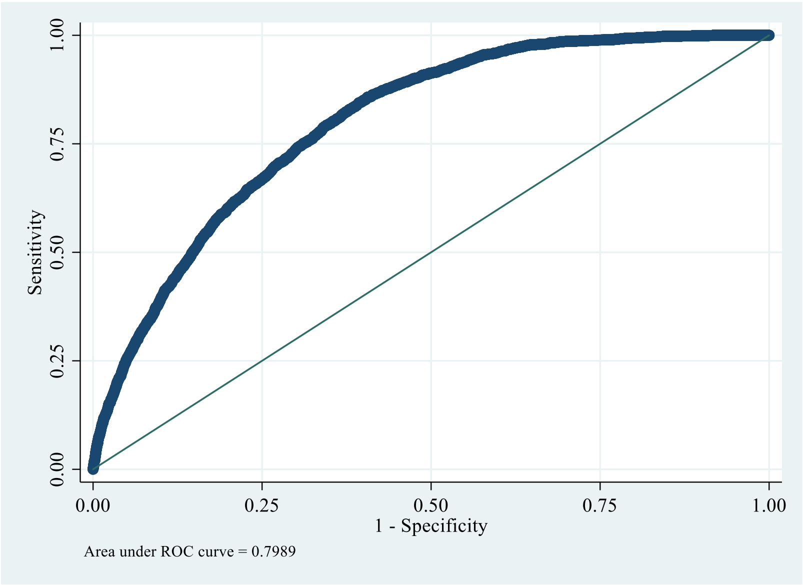

b; Outcome: Cognitive impairment

Figures S2a and S2b: Plots of residuals against fitted values (close to zero variation of mean of the residuals (y-axis) against fitted values (x-axis) of predictors suggests no violation of linearity)

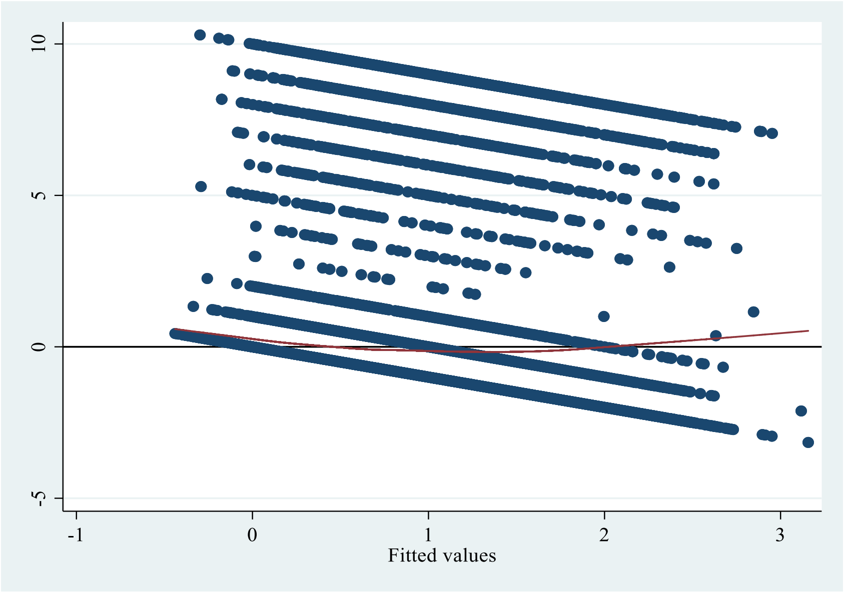

a; Outcome: Depressive symptoms

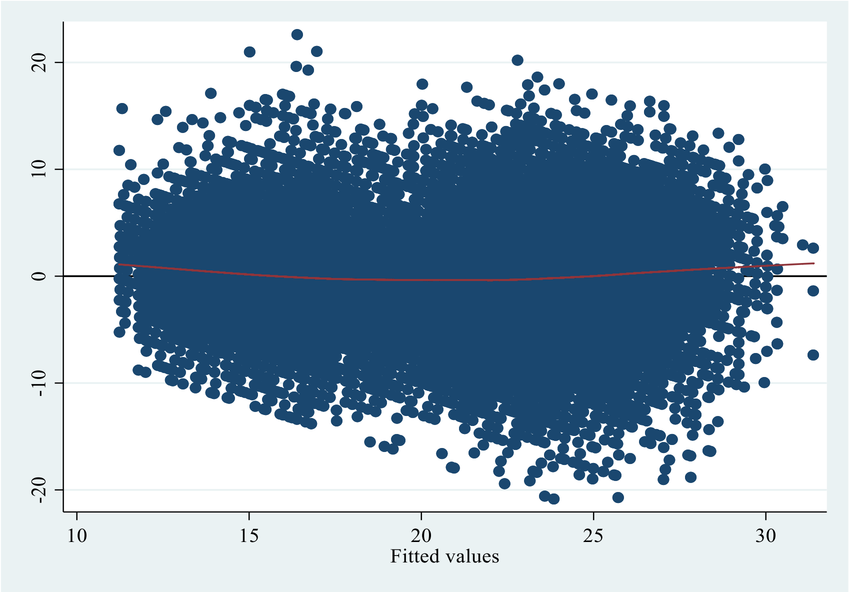

b; Outcome: Cognitive impairment

Figures S3a and S3b: Quantile-quantile plots showing the normality of the residuals

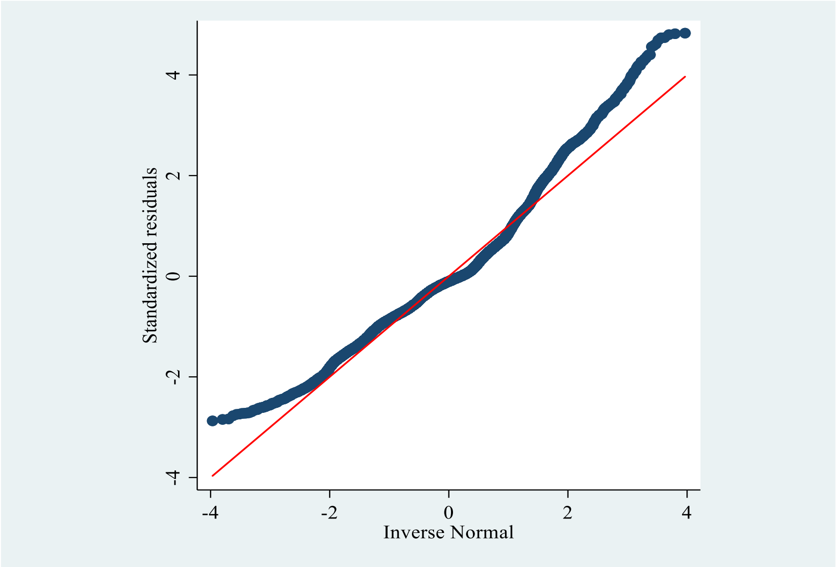

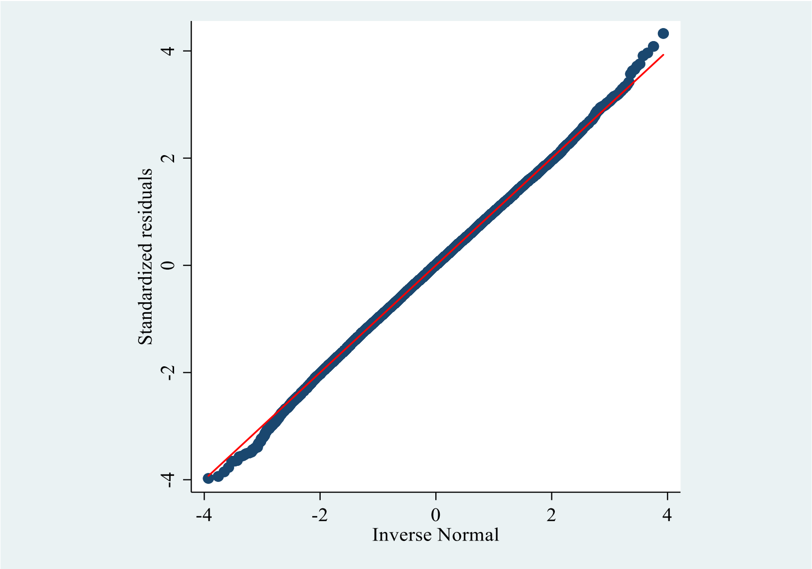

a; Outcome: Depressive symptoms

b; Outcome: Cognitive impairment

Table S1: VIF estimates for the selected explanatory variables

Variables	Sub-categories	VIF	1/VIF
**Age (years)**	60-69		
	70-79	1.09	0.918416
	80+	1.12	0.89233
**Sex**	Male		
	Female	1.29	0.773771
**Marital status**	Currently in union		
	Widowed	1.54	0.649921
	Others	1.06	0.939649
**Living arrangements**	Living alone		
	With spouse	4.98	0.200711
	Others	4.57	0.219032
**Educational status**	No/ primary		
	Secondary	1.23	0.812354
	Higher	1.3	0.767886
**Self-rated health**	Good		
	Poor	1.04	0.962564
**MPCE quintile**	Poorest		
	Poorer	1.65	0.606985
	Middle	1.69	0.590296
	Richer	1.74	0.574321
	Richest	1.87	0.533453
**Religion**	Hindu		
	Muslim	1.07	0.931678
	Others	1.39	0.720581
**Caste**	SC/ST		
	OBC	1.64	0.610384
	Others	1.72	0.580249
**Place of residence**	Urban		
	Rural	1.23	0.810669
**Region**	North		
	Central	1.62	0.618253
	East	1.67	0.5983
	Northeast	1.64	0.609578
	West	1.97	0.50786
	South	1.47	0.678217
Mean VIF		1.73	
